# RF1 attenuation enables efficient non-natural amino acid incorporation for production of homogeneous antibody drug conjugates

**DOI:** 10.1038/s41598-017-03192-z

**Published:** 2017-06-08

**Authors:** Gang Yin, Heather T. Stephenson, Junhao Yang, Xiaofan Li, Stephanie M. Armstrong, Tyler H. Heibeck, Cuong Tran, Mary Rose Masikat, Sihong Zhou, Ryan L. Stafford, Alice Y. Yam, John Lee, Alexander R. Steiner, Avinash Gill, Kalyani Penta, Sonia Pollitt, Ramesh Baliga, Christopher J. Murray, Christopher D. Thanos, Leslie M. McEvoy, Aaron K. Sato, Trevor J. Hallam

**Affiliations:** Sutro Biopharma Inc, South San Francisco, CA 94080 USA

## Abstract

Amber codon suppression for the insertion of non-natural amino acids (nnAAs) is limited by competition with release factor 1 (RF1). Here we describe the genome engineering of a RF1 mutant strain that enhances suppression efficiency during cell-free protein synthesis, without significantly impacting cell growth during biomass production. Specifically, an out membrane protease (OmpT) cleavage site was engineered into the switch loop of RF1, which enables its conditional inactivation during cell lysis. This facilitates extract production without additional processing steps, resulting in a scaleable extract production process. The RF1 mutant extract allows nnAA incorporation at previously intractable sites of an IgG1 and at multiple sites in the same polypeptide chain. Conjugation of cytotoxic agents to these nnAAs, yields homogeneous antibody drug conjugates (ADCs) that can be optimized for conjugation site, drug to antibody ratio (DAR) and linker-warheads designed for efficient tumor killing. This platform provides the means to generate therapeutic ADCs inaccessible by other methods that are efficient in their cytotoxin delivery to tumor with reduced dose-limiting toxicities and thus have the potential for better clinical impact.

## Introduction

Consistent and robust production processes for the site-specific generation of antibody-drug conjugates (ADCs) have the potential to generate therapeutic products comprised of a single molecular entity rather than the heterogeneous mixtures present in the approved products of today (Adcetris^1^ and Kadcyla^2^). The potential benefit is homogeneous ADC products that display more efficient tumor killing (more potency with greater tolerability) than their heterogeneous counterparts. Efficient incorporation of specifically conjugatable non-natural amino acids (nnAAs) into antibodies is an attractive approach to generating homogeneous ADCs offering great flexibility in where they can be positioned. Combining use of nnAAs with cell-free protein synthesis provides a means to rapidly express and discover optimal conjugations sites for tumor cell killing and tolerability assessments and offers opportunities for efficient production.

Multiple systems for the incorporation of nnAAs into proteins have been previously exemplified, and in particular suppression of the TAG stop codon (amber) has been widely used^3^. Similarly, we recently reported that our cell-free *in vitro* transcription-translation platform, Xpress CF^4^, employs an engineered orthogonal aminoacyl tRNA synthetase (aaRS) that enables incorporation of either *para*-azidophenlyalanine (pAzF) or *para*-azidomethylphenylalanine (pAMF) non-natural amino acids (nnAAs) into aglycosylated IgGs^5^. As with other amber suppression systems^6–11^, we observe that competition with endogenous release factors (RFs) limits the extent of nnAA incorporation into many sites. Specifically, RF1 undergoes a large conformational switch upon binding to the ribosome, where it competes with the orthogonal tRNA_CUG_ for UAG stop codons, blocking pAzF or pAMF incorporation, and terminating protein chain elongation^12^. Thus, expression efficiency and titers can be significantly impacted when incorporating nnAAs compared to wild-type protein expression.

To address this limitation, several groups have demonstrated that attenuating RF1 activity in cell-free systems improves nnAA incorporation. As RF1 was once thought to be an essential protein, critical for cell growth^13^, many indirect approaches to inactivate RF1 in cell-free expression systems were first employed, including using inhibiting antibodies^6^ or RNA aptamers^7^, RF1 depletion by subtractive affinity chromatography^8^, or excluding RF1 from recombinantly reconstituted cell-free systems (i.e. PURE)^9^. However, these approaches add significant process requirements and cost to generate an RF1 attenuated cell-free protein production system. More recent work has shown that RF1 can indeed be removed from certain strains of *E. coli*, provided that RF2 expression is increased by removing the RF2 autoregulation +1 frameshift and “fixing” the A246T mutation – though this generally leads to a slower growth rate of the engineered cells^10^. RF1 can also be eliminated from MDS42^10^, a minimal *E. coli* strain that has almost 700 genes deleted. Others have shown that RF1 can also be knocked-out, provided that TAG stop codons are replaced with TAA, allowing RF2 to essentially replace the need for RF1^11, 14^.

In our cell-free antibody production system, engineered *E. coli* strains are used to provide an “extract”, the processed biomass raw material that contains all of the necessary components for efficient cell-free transcription, translation and antibody assembly. We sought an alternative, and simple solution for RF1 inactivation that could be readily applied to those strains at production scale. To maintain scalability of our system, it was crucial not to compromise growth rate, which is important for extract activity^15^, nor to incur any additional processing steps or costly additives, such as inactivating antibodies to RF1.

In this study we demonstrate that the *prf*A gene of *E. coli* that codes for RF1 can be reengineered to code for a mutant RF1 (RF1^MUT^) that is sensitive to OmpT protease cleavage, allowing normal cell growth rates for highly active extract production. By design, RF1^MUT^ is clipped and inactivated upon exposure to OmpT, which is localized on the outer cell-membrane and therefore not in contact with intracellular proteins, like RF1, prior to cell lysis. This engineered, scalable, cell-free *in vitro* transcription-translation platform, termed Xpress CF+, enables nearly uniform nnAA incorporation across sites, and is a further evolution of our Xpress CF platform with which we previously reported production of site-specific ADCs. Site-specific conjugation can then be performed using bio-orthogonal, strain-promoted alkyne-azide cycloaddition (SPAAC or “copper-free click chemistry”) using dibenzocyclooctyl (DBCO) functionalized cytotoxin to generate homogenous ADCs^5, 16, 17^. Here we demonstrate that our improved Xpress CF + system now enables nnAA incorporation at previously intractable sites on both the heavy chain (HC) and light chain (LC) of an IgG1. We show that several previously inaccessible ADCs can now be made, including ones with higher drug to antibody ratios (DARs) by incorporating multiple nnAAs into the same polypeptide chain. Moreover, we find one site in particular, (HC F404) results in increased thermal stability and also allows for enhanced drug-linker stability *in vivo* – coincidentally, this particular site required RF1 attenuation to allow for nnAA insertion, and was therefore only enabled and discovered by the Xpress CF+ system.

## Results

### TAG Suppression Efficiency Varies with Site

**Figure 1:**
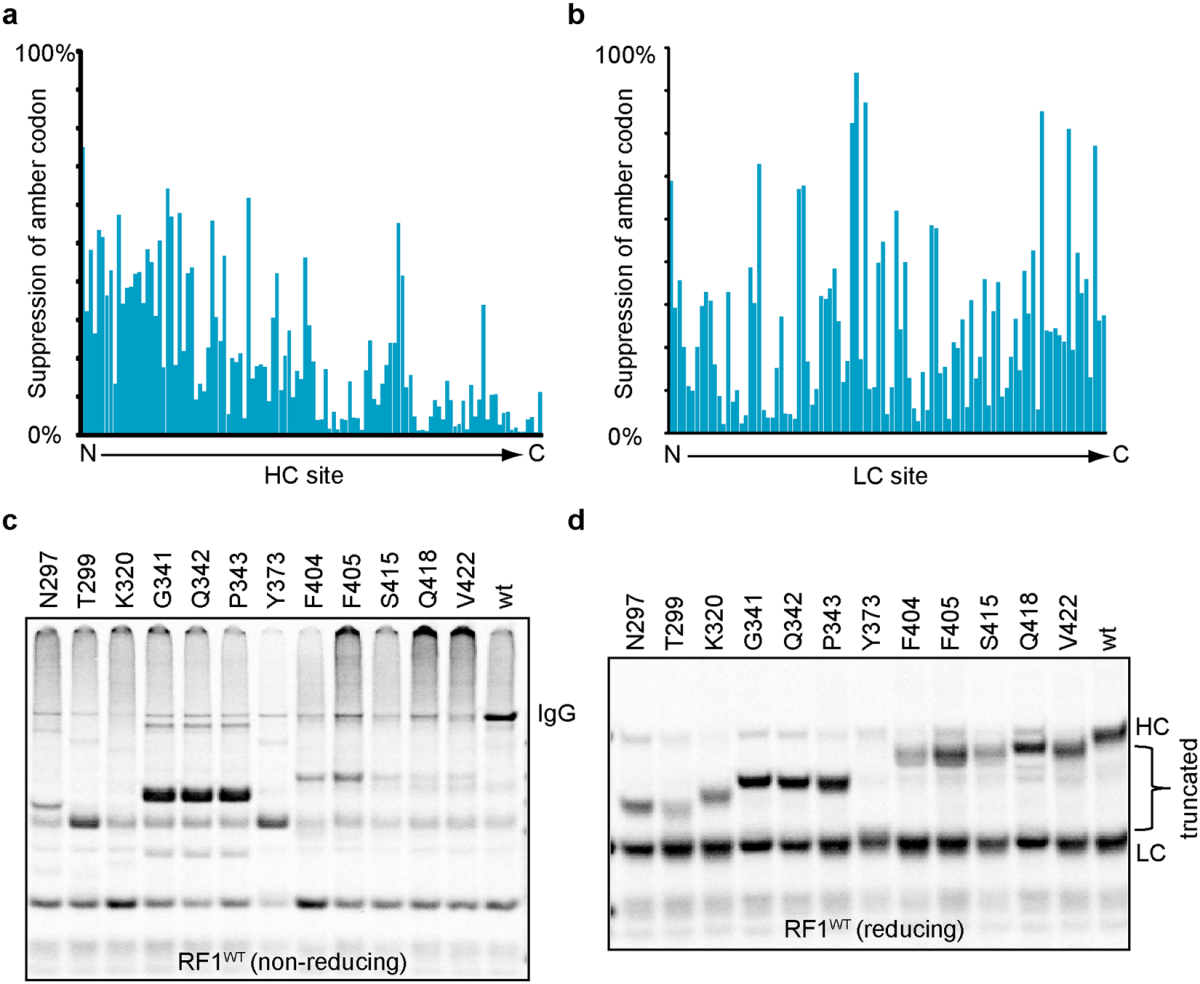
TAG suppression efficiency in RF1^WT^ extract varies with site. (**a**) Suppression efficiency of the amber (TAG) codon at the sites in heavy chain substituted with pAMF declines in general from N- to C-terminus in RF1^WT^ extract. The suppression efficiency at a site is calculated as a percentage of unsubstituted HC band intensity in a ^14^C reducing autoradiography. (**b**) Suppression efficiency of the TAG codon in the light chain is unpredictable and many sites are intractable in RF1^WT^ extract. (**c**) 12 sites in HC with very low suppression efficiency in RF1^WT^ extract are shown on non-reducing autoradiography. IgG yields were extremely low in the RF1^WT^ extract due to chain truncation. (**d**) The same 12 sites as in panel b are shown in reducing autoradiography.

After analyzing available crystal structures of the trastuzumab Fab (PDB code 1N8Z) and Fc domain (PDB code 1FC1), we identified solvent accessible sites as well as some inaccessible control sites on the IgG away from the CDRs (PDB code 1HZH). To examine how nnAA incorporation efficiency varies as a function of site, we mutated codons corresponding to these 213 sites to TAG codons on the HC and LC of aglycosylated trastuzumab. Our results suggest that nnAA incorporation, i.e. the TAG suppression efficiency, varies significantly by site in an IgG (Fig. 1a,b, Table S1a,b). Generally, suppression efficiency declines from the N to C terminus on the HC, though there is unpredictable variation at each site that deviates from this trend. The 12 least suppressed sites in the HC are shown as an example to illustrate the poor suppression efficiency compared to wild-type (Fig. 1c,d). Thus, suppression efficiency places an inherent limitation on high yield expression of many nnAA containing antibodies, which in turn restricts what site specific ADCs can be produced, regardless of whether or not their drug-like properties would prove superior.

### Engineering Conditionally Inactive RF1 Mutant (RF1^MUT^)

**Figure 2:**
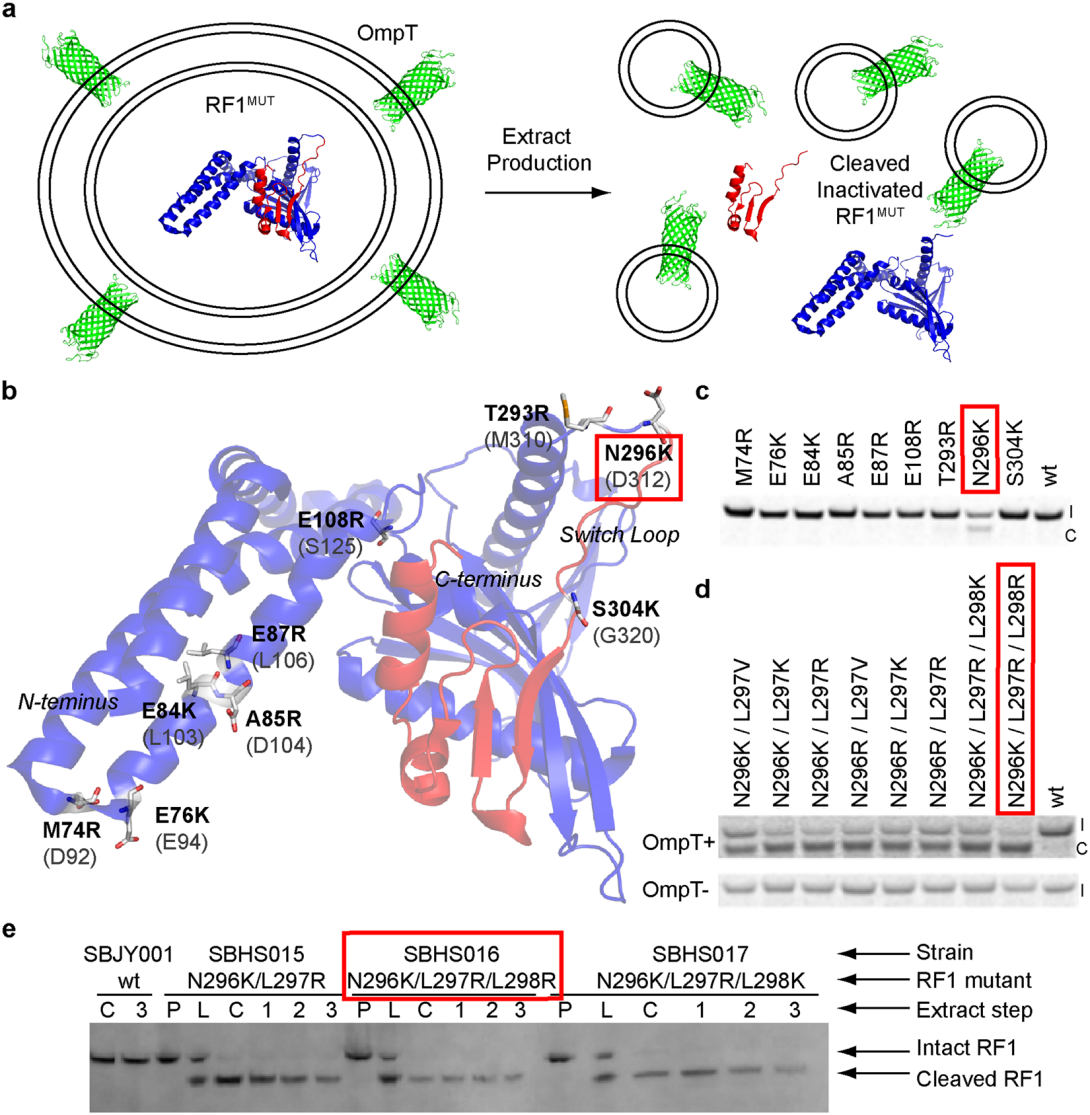
Engineering a contionally inactive RF1^MUT^ strain. (**a**) OmpT protease is sequestered on the cell surface preventing cleavage of RF1^MUT^ which allows normal cell growth during fermentation. During extract production, cell lysis exposes OmpT to intracellular proteins which cleaves and inactivates the engineered RF1^MUT^. (**b**) The initial mutations (bold) made to RF1 are highlighted on the RF2 crystal structure (PDB code 1GQE; cartoon model; RF2 residues in parenthesis). Though the RF1 structure was available (PDB code 2B3T), the RF2 structure was used since it has an ordered switch loop and ~40% identity to RF1. Non-conserved sites adjacent to basic residues were chosen for point mutations which enables incorporation of canonical dibasic OmpT cleavage sites with minimal change to the native RF1 sequence and activity. (**c**) The designed RF1 mutants were expressed in small-scale cell-free reactions to screen for proteolytic susceptibility. Only the N296K mutant in the switch loop showed OmpT sensitivity. (I = intact; C = cleaved) (**d**) To improve proteolytic cleavage upon cell-lysis, additional basic residues were added to the switch loop of the N296K mutant. Cell-free reactions were run in OmpT+ and OmpT− extract to demonstrate that proteolysis is dependent on OmpT. (I = intact; C = cleaved) (**e**) Three separate genomic RF1 mutants were engineered using OMAR starting from parent strain SBJY001 (RF1^WT^). A Western blot shows RF1 cleavage occurs during extract production immediately after cell lysis for each mutant strain. No RF1 cleavage is observed in the SBJY001. (P = cell pellet after fermentation, L = cell lysis, C = extract clarification by centrifugation, and 1–3 = incubation time in hours after clarification).

To enable RF1 to remain active during normal cell growth and become inactivated only during extract production, we engineered in OmpT cleavage sites. Since OmpT is sequestered on the cell surface, we reasoned it would be unable to cleave RF1 during normal cell growth. However, cell lysis exposes OmpT to intracellular proteins allowing cleavage and inactivation of RF1 during extract preparation (Fig. 2a). To minimize the impact of mutations on RF1 function before cleavage, non-conserved residues adjacent to existing basic residues in RF1 were targeted first. Thus, single point mutations to R or K yielded canonical OmpT dibasic cleavage motifs^18^. An initial *in vitro* screen in OmpT + extract showed cleavage of the N296K mutant, but not any of the other sites (Fig. 2b,c). We note that N296K is located in the switch loop of the RF1 structure, which is likely to be more proteolytically susceptible since it lies in a disordered loop^19^. Moreover, the switch loop is necessary for RF1 function, so it was reasoned that cleavage should inactivate RF1^20^.

Since cleavage was incomplete with the single N296K substitution, we made additional mutations to increase its efficiency. The addition of one more basic residue (N296K/L297R and N296K/L297K) increased cleavage, and two more basic residues (N296K/L297R/L298K and N296K/L297R/L298R) further increased cleavage *in vitro* (Fig. 2d). Three RF1 variants were engineered into the parent strain SBJY001 (A19 ∆endA ∆tonA ∆speA ∆tnaA met+ ∆sdaA ∆sdaB ∆gshA ∆gor) using Oligonucleotide Mediated Allelic Replacement (OMAR)^21^ yielding strains SBHS015 (N296K/L297R), SBHS016 (N296K/L297R/L298R), and SBHS017 (N296K/L297R/L298K). Small scale extract production using each of the three strains showed RF1 cleavage immediately after cell lysis as intended (Fig. 2e). As a control, extract was also made using the parent SBJY001 strain, which showed wt RF1 remains intact throughout the process. The growth rates of SBJY001 and three RF1^MUT^ strains, SBHS015, 016 and 017 were similar at roughly 0.6 hr^−1^ (Fig. S1), suggesting the RF1 mutation does not affect cell growth and presumably should not affect cell extract activity. SBHS016 showed greater proteolytic cleavage than SBHS015 and SBHS017. Hence, it was chosen for large scale extract preparation and further testing.

### RF1^MUT^ Extract Improves TAG Suppression Efficiency

**Figure 3:**
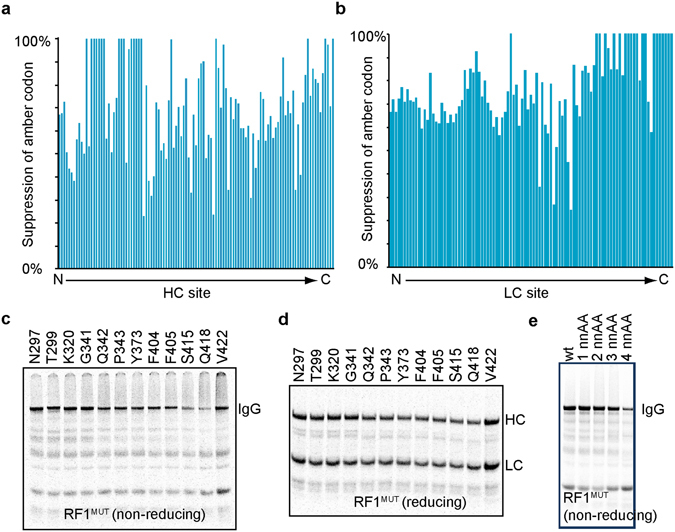
RF1 mutant extract improves nnAA incorporation. (**a**,**b**) The amber suppression efficiency increases in HC and LC across most sites in the RF1 mutant extract. (**c**) The non reducing autoradiography shows the efficient incorporation of pAMF at previously intractable sites using the RF1^MUT^ extract. (**d**) The reducing autoradiography indicates the suppression efficiency of amber codon in RF1^MUT^ is greatly improved with minimal truncation products. (**e**) RF1 mutant extract enables incorporation of multiple nnAAs. No increase in truncation prodcts was observed and up to 3nnAAs were incorporated with little loss in expression titer as compared to WT trastuzmab. 1 nnAA = F404, 2 nnAA = F404 + Y180, 3 nnAA = F404 + Y180 + A118, 4 nnAA = F404, Y180, A118, V5, all sites are on HC.

A second experiment in SBHS016 (RF1^MUT^) extract was performed to assess overall TAG suppression efficiency across a range of sites. Importanly, the 12 sites that showed the poorest suppression efficiency with the original extract were included to enable direct comparison to the first nnAA scan. The IgG titers containing nnAA at these previously intractable sites increased substantially with no significant HC truncation products observed (Fig. 3d). Additional sites were included in the second study (e.g. buried, non-surface accessible residues) to get a better idea of overall suppression efficiency. In RF1^MUT^ extract, the amber suppression efficiency is high for both the HC and LC across most sites (Fig. 3a,b, Table S2a,b). Moreover, titers of nnAA-containing IgGs increased significantly (Fig. 3c) and are comparable to the titer of wild type IgG in RF1^WT^ extract in many cases. The yields of wild type IgG and well suppressed nnAA containing IgGs in the high throughput screening mode were around 200–300 ug/mL.

### Conjugation Site Affects *In Vivo* Half-Life, Linker Stability, and Efficacy

**Figure 4:**
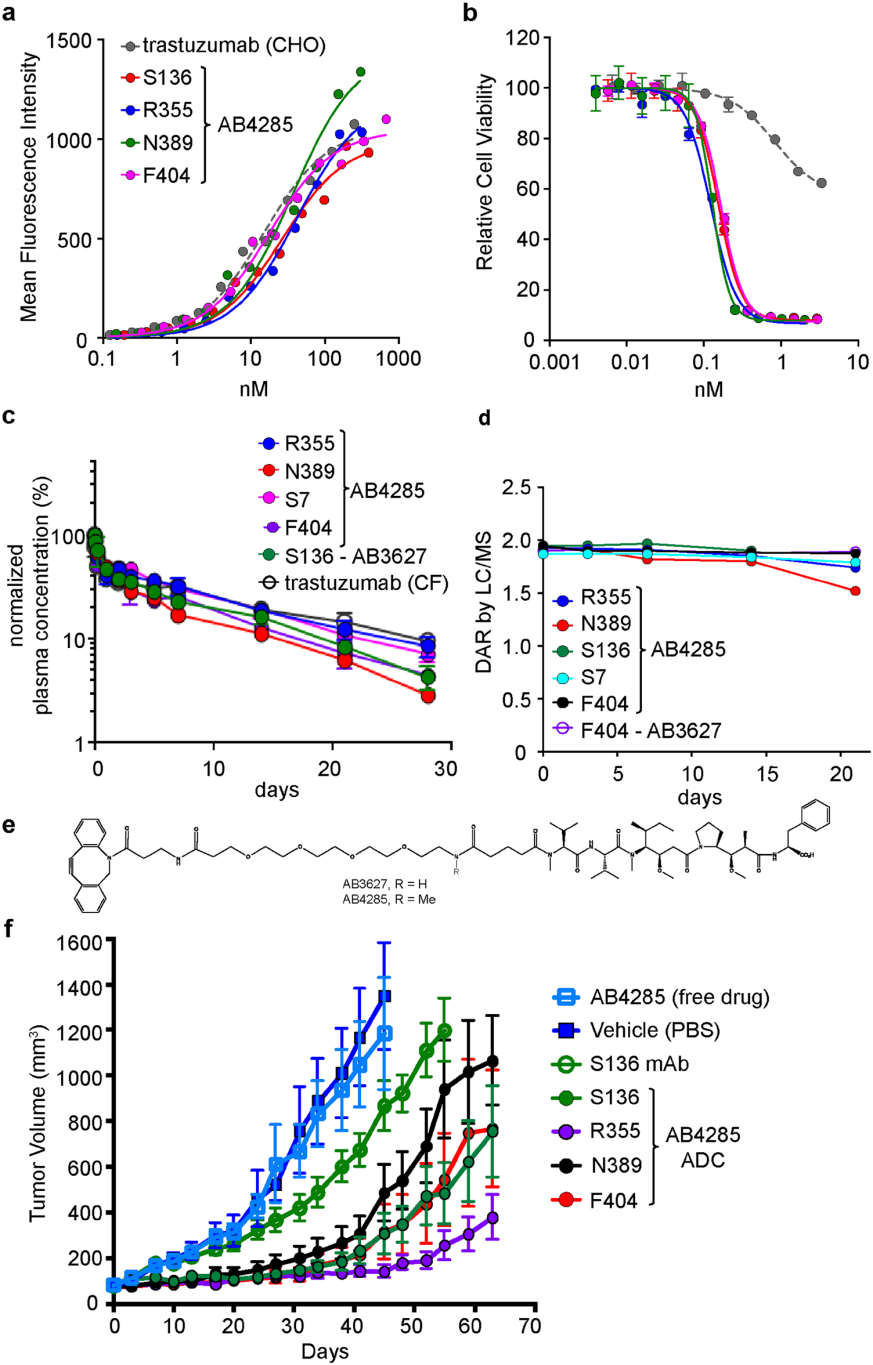
Site-specific ADCs cell binding, cell killing, and plasma stability. (**a**) Cell binding of four site-specific ADCs. All ADCs show similar *in vitro* cell binding to Her2 + SKBR3. (**b**) Cell killing of four site-specific ADCs. All ADCs show similar cell killing activities to Her2 + SKBR3. (**c**) Pharmacokinetics of four site-specific ADCs. Beige nude XID mice were dosed with 2–5 mg/kg of conjugate and plasma samples were collected and analyzed by Her2 ECD ELISA to determine total antibody concentrations over time. (**d**) Drug-linker stability was assessed by LC-MS. (**e**) Structures of DBCO-MMAF drugs-linkers. AB4285 is a methylated derivative of AB3627. (**f**) Site-specific ADCs *in vivo* efficacy. KPL-4 orthoptic Beige nude xid mice tumor models. Animals were dosed with a single i.v. injection of trastuzumab-DBCO MMAF site specific ADCs (15 mg/kg, n = 10 mice per group) and tumor growth was monitored over 60 days. Transient regression followed by tumor regrowth after single treatment was site dependent. Efficacy was compared with unconjugated trastuzumab control produced in cell free (S136 mAb), vehicle (PBS) and drug-linker at equivalent dose. Multiple dosed glycosylated, CHO-produced trastuzumab served as a positive control.

Many cell-free produced, aglycosylated trastuzumab (trastuzumab^CF^) site-specific ADCs with excellent suppression efficiencies were assayed *in vitro* for DAR, cell killing activity, and thermal stability. Several of the best ADCs were chosen for expression scale-up using RF1^MUT^ extract and characterization *in vivo*. In particular, we sought to determine how drug conjugation site would affect stability and *in vivo* pharmacokinetics. Thus, monomethyl auristatin F (MMAF) was conjugated to HC sites S136, R355, N389, F404 and LC S7; F404 was among the previously intractable sites. Moreover, MMAF was conjugated to trastuzumab through different linkers to assess the effect of linker structure *in vivo* (AB4285 and AB3627, structures are shown in Fig. 4e). All of the trastuzumab^CF^ nnAA variants show high expression and recoveries comparable to WT trastuzumab^CF^ using RF1^MUT^ extract. All five sites show high conjugation efficiencies as measured by LC/MS (Table 1). The cell binding affinity of these ADCs on SKBR3 cell are all comparable to CHO-expressed trastuzumab and show high cell killing potency (Fig. 4a,b and Table 1). HC F404 ADC even shows improved thermal stability post conjugation (Table 1).

**Table 1:** DAR, SKBR3 cell binding Kd, cell killing IC50 and melting temperature (Tm) of ADC variants.

Samples		DAR by LC/MS	SKBR3 cell binding, Kd (nM)	SKBR3 cell killing, IC50 (nM)	Ab only		ADC	
					Tm1 °C	Tm2 °C	Tm1 °C	Tm2 °C
Trastuzumab (CHO)		—	2	—	61.5	76.8	—	—
Trastuzumab^CF^ MMAF (AB4285)	LC-S7	1.85	7.6	0.11	62.0	76.9	60.7	76.2
	HC-S136	1.84	4.7	0.15	61.4	76.7	61.1	76.3
	HC-R355	1.97	5.6	0.12				
	HC-N389	1.96	7.2	0.13				
	HC-F404	1.97	2.4	0.17	61.2	76.6	63.5	76.5
Trastuzumab^CF^ Maytansine (SC-236)	HC-F404	1.77	n/a	0.051	61.2	76.6	n/a	n/a
	HC-F404 Y180	3.83	n/a	0.031	n/a	n/a	n/a	n/a
	HC-F404 Y180 A118	5.82	n/a	0.051	n/a	n/a	n/a	n/a
	HC-F404 Y180 A118 V5	7.43	n/a	0.084	n/a	n/a	n/a	n/a

Immunodeficient Beige Nude XIDs or Balb/c mice were used to study the pharmacokinetics properties of these ADCs. Mice were injected with a single i.v. dose of 2 mg/kg of each ADC and plasma samples of terminal bleeds were harvested at multiple time points up to 28 days. Plasma samples were analyzed for total circulating trastuzumab^CF^ antibody concentrations using anti-Her2 ELISA. Two compartmental pharmacokinetic analysis of single site specific ADCs suggested that clearance (CL) of the trastuzumab^CF^ with nnAA incorporated at different sites was comparable across several sites, namely HC S136, R355, F404 and LC site S7. HC N389 showed faster clearance demonstrating that conjugation site affects PK (Fig. 4c). In general, the different linkers AB4285 and AB3627 did not affect the PK profiles of the antibody.

To assess the plasma stability of the linkers, samples from the PK study were analyzed using a bead-based affinity capture and LC/MS (Fig. 4d). We first evaluated the *in vivo* stability of the AB3627 drug-linker that was conjugated to sites HC-S136 and HC F404. AB3627 conjugated to HC site S136 show significant loss of drug on day 7 in Balb/c mouse (Fig. S2). S136 conjugated to AB4285, which is a methylated derivative of AB3627, shows improved stability in Beige nude XID mouse. Similarly, AB4285 conjugated to trastuzumab^CF^ at HC site R355, F404 and LC site S7 show high drug-linker stability in circulation up to day 21. A modest decline in stability was observed for AB4285 conjugated to HC site N389. Remarkably, AB3627 conjugated to HC F404 shows stability up to day 21, suggesting that local environment around F404 protects this otherwise labile linker from degradation *in vivo*.

**Figure 5:**
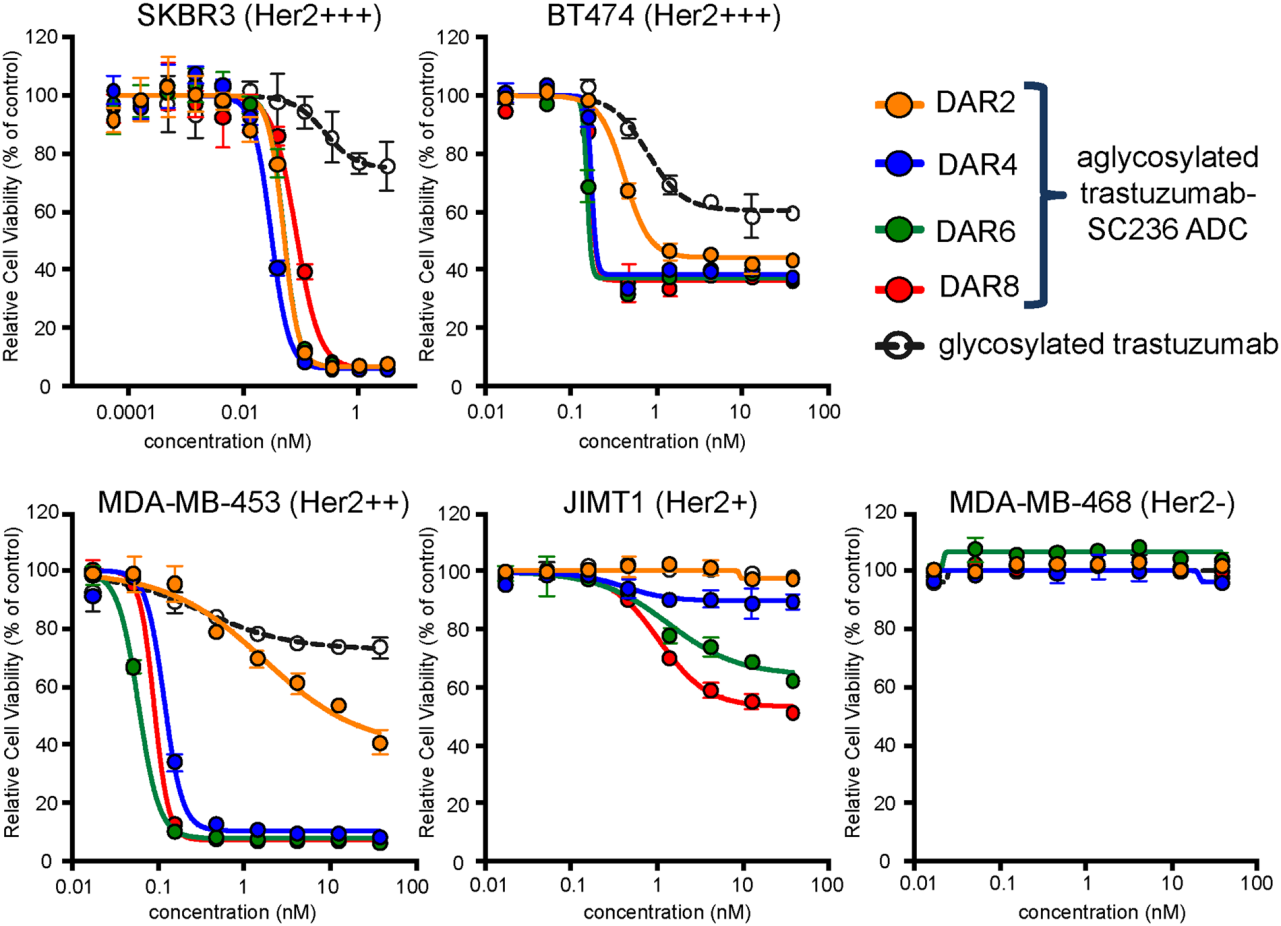
Improved potency by increasing ADC DAR. ADCs withs DAR 2, 4, 6 and 8 (see Table 1 for positional combination variants) were tested on SKBR3 (1.25 K cells), BT474 (1.875 cells), MDA-MB-45 (1.875 cells), JIMT1 (1.25 K cells) and MDA-MB-468 (1.875 K cells) lines (5 day treatment). ADCs with different DARs showed similar cell killing activites on high Her2 SKBR3 cells. DAR 2 ADC showed less killing activity on moderate Her2 BT474 and MDA-MB-453 cells, while DAR 4, 6, 8 ADCs showed similar cell killing activities. The cell killing activites with different DARs differentiate on Her2 low JIMT1 cells. No cell killing activiy was observed on Her2 negative MDA-MB-468 cells.

To determine *in vivo* efficacy, the same site-specific ADCs were evaluated in a KPL-4 orthotopic animal model^2, 22^. KPL-4 tumors (~100–150 mm^3^) grown in the mammary fat pads of Beige Nude Xid mice and treated with single i.v. dose of 15 mg/kg of each ADC. All four site-specific ADCs tested show significantly better efficacy in inhibiting KPL-4 tumor growth measured over 40 days, compared with unconjugated trastuzumab^CF^, vehicle and free drug (*P* < 0.001) (Fig. 5f, Table S3). Notably, we find that conjugation to R355 is significantly more efficacious than conjugation to N389 at day 63 (*P* < 0.05). Poor efficacy of the HC N389 ADC is consistent with its higher clearance and poor drug-linker stability in circulation (Fig. 5c). Though other sites show differences in efficacy, we find they are not statistically significant (e.g. conjugation to R355 vs F404 *P* = 0.3676 at day 63, Table S4). The unconjugated, aglycosylated trastuzumab^CF^ shows minimal efficacy, as is expected due to its lack of effector function.

### RF1^MUT^ Extract Enables Production of ADCs with Higher DAR leading to higher potency

We also examined the impact of the RF1 attenuation in RF^MUT^ extract on the ability to incorporate multiple nnAAs in the same polypeptide chain. Specifically, we layered multiple nnAA incorporation sites onto one another on the HC in the following order: F404, Y180, A118, V5 and assessed the expression of the resultant constructs. In contrast to RF1^WT^ extract, where even incorporation into the single F404 site resulted in a majority of truncated product (Fig. 1c), RF1^MUT^ extract allowed the incorporation of up to 4 nnAAs; with little impact on expressing titers observed up to 3nnAAs (Fig. 3e). It is unclear whether the observed drop in expression titers for the 4nnAA construct is related to cumulative suppression efficiency issues, or simply stability of the resultant nnAA containing trastuzumab. Since even in the construct containing 4nnAAs, no significant truncation products were observed by autoradiography, the latter option seems probable. Thus, RF1^MUT^ extract efficiently enables the production of IgGs containing multiple nnAAs, which in turn enables the production of higher DAR ADCs that can deliver more cytotoxic drug to the targeted cells, in theory increasing efficacy.

To investigate the impact of DAR on the potency of our site specific ADCs we also conjugated DBCO-PEG4-maytansine (SC-236) to these multi-nnAA containing trastuzumab variants. The resulting ADCs had theoretical DAR values of 2, 4, 6 and 8 respectively, with the actual DARs of the constructs shown in Table 1. The LC-MS analysis of conjugates is shown in Fig. S3. Specifically, we tested these different constructs against a panel of Her2 expressing cell-lines – i.e. SKBR3, BT474, MDA-MB-453, and JIMT1 – as well as the Her2 negative cell-line MDA-MB468. We observed that depending on the cell-line, DAR of the ADC impacts cell-killing potency differently (Fig. 5). In the case of SKBR3 cells, which show high sensitivity to our maytansine based trastuzumab ADCs, very little effect is seen as DAR is increased. Conversely, MDA-MB-453 cells are only potently killed with ADCs that have DARs of 4 or higher. Generally across cell-lines, however, a trend is observed that higher DARs result in higher potency ADCs. Also of note is that even at DARs of 8, the ADCs do not seem to affect Her negative cells.

## Discussion

Two recently approved therapeutics, Adcetris/brentuximab vedotin/SGN-35 and Kadcyla/trastuzumab emtansine/T-DM1, exemplify alternative viable commercial paths for ADCs. Both ADCs^23, 24^ are made through the conjugation of microtubule inhibitors to either cysteines (SGN-35) or lysines (T-DM1) that are natively part of the antibody. The resulting ADCs are therefore heterogeneous mixtures that display a distribution of site and number of cytotoxic drug conjugation^25^. Each of the individual species, present in the mixture, is expected to have distinct biophysical properties and thus exhibit separate pharmacological properties; though characterization of each species remains challenging. Hence, many groups have turned to homogeneous, site-specific ADCs, which can be specifically tuned and characterized in depth; the promise being that this allows for the selection of superior biophysical and therapeutic properties. Indeed, we show that we can optimize conjugation site(s) as well as DAR by leveraging the RF1^MUT^ extract in our cell-free expression system.

Several previous studies and the work presented here make it clear that ADCs with defined conjugation sites and DARs affect important drug attributes such as homogeneity, stability, PK, and toxicity^26–35^. Because our linkage is based on copper free click chemistry using a cyclooctyne moiety on the drug molecule and an azide on the non-natural amino acid incorporated into the antibody scaffold, it was expected that the yield of the specific positional variant would depend on (a) the ability to incorporate the non-natural amino acid into a specific position during translation in the cell-free system (b) the ability to properly fold the heavy or light chain variant incorporating the nnAA at that specific position (c) the ability to assemble the properly folded LC and HC into a fully functional antibody and (d) the accessibility of the azide moiety on the fully assembled antibody to the cyclooctyne to enable efficient 1,3-dipolar cycloaddition.

However, we are encoding a single amino acid, so it is easy to place it any position in the antibody without disrupting its structure lending us more freedom to design optimal ADCs than other technologies that rely on larger peptide motifs^34^. The azide handle is also completely bio-orthogonal, so we do not have to contend with glutathione adducts formed from engineered thiomabs^26^. This prevents the need to process the mutant mAb by selective reduction enabling conjugation to mAbs prepared using standard purification procedures. Using the RF1^MUT^ extract also enables suppression efficiencies to be elevated, which unlocked previously intractable sites for the incorporation of nnAAs compared to other technologies that contain wild-type RF1^30^. Moreover, this also allows us to readily incorporate multiple nnAAs into the same polypetide for the production of higher DAR ADCs.

Using our platform, top conjugation sites were selected for *in vivo* characterization, based on initial screening for the following criteria *in vitro*: good suppression efficiency, correct IgG assembly, and high conjugation efficiency while maintaining properties of antigen binding, cell killing and thermostability. We observed early on, that inefficient suppression of the TAG codon for nnAA incorporation can greatly limit titer, and hence the ability to practically utilize certain sites for nnAA incorporation. As others have shown, we also observe that several *in vivo* pharmacological properties of these ADCs are site-dependent^26, 28, 36^. In particular, the efficacy of ADCs in our tumor xenograft models depends on the drug conjugation site, as we see a span of activities for these four conjugates. In particular, R355 is significantly more potent than N389. We see a site-dependence in clearance rate, as N389 clears significantly faster than the other conjugates, though all conjugates show similar half-lives as the parent unconjugated antibodies. Though R355 appears slightly more potent than F404 and S136, the differences are not statistically significant, which is consistent with their similar PK profiles and *in vitro* activities. Conjugation site also affects linker degradation *in vivo* as N389 loses drug in plasma, but the other AB4285 conjugates retain the same DAR for 20 days. Notably, the un-methylated amide linker AB3627 was found to be more degradation prone, except when conjugated to F404.

Overall this suggests that F404 is an excellent site to minimize unwanted *in vivo* degradation. F404 resides on the CH3 constant domain, directed to the pocket between the CH2 domains that is normally glycosylated on mammalian-produced antibodies. Thus, the linker and drug might be uniquely protected from exposure when conjugated F404. Moreover, we note that conjugation to F404 actually enhances the thermal stability of the resulting ADC. This might also be due to filling the empty glycosylation pocket, or because of the antibody is simply stabilized by the presence of the hydrophobic pAMF and DBCO moieties at this particular site. F404 has the most hydrophobic local environment of the four tested sites, suggesting it might be most compatible with the conjugation chemistry employed. Interestingly, the thermal stability does not necessarily translate to significantly improved pharmacokinetics as other conjugates show comparable half-life (e.g. R355 and S7).

The ability to conjugate to F404 was specifically enabled by the engineering of our new RF1^MUT^ strain, as our initial extract showed very low suppression efficiency at this site. In general, the RF1^MUT^ extract shows significantly improved incorporation of nnAA throughout the IgG HC and LC without many truncation products. Most sites show similar titers to the wild type proteins, but not all sites are expressed to the same high level. This suggests there are additional factors that limit suppression efficiency, e.g. mRNA secondary structure might affect suppressor tRNA recognition. Or ribosomes stalled at certain TAG sites might be shunted to the tmRNA/ssrA trans-translation degradation pathway^37^. Nonetheless, this strategy for inactivating RF1 proved crucial in enabling a wider variety of ADCs to be produced, either by unlocking previously intractable sites, or allowing for higher DAR ADCs. This in turn broadens the possible choice of ADCs to be made, tested and interrogated for function and potency, which has the potential for enabling the discovery of highly optimized ADCs that are tailored in great detail, in terms of DAR, site combinations, linkers, and drugs, to deliver the best possible therapeutic modality for a particular indication.

The xenograft data shown here suggest that position of conjugation has marked effect on tumor killing, even when stability and pharmacokinetics appear equivalent. It will be intriguing to see whether these differences also improve or maintain tolerability and show improved therapeutic index in the clinic.

Development of these new RF1^MUT^ strains opens the way for scaleable and efficient production processes for nnAA containing proteins. RF1^MUT^ strains may now form the basis of cell-free cGMP processes for clinical trial material and, potentially, a preferred commercial production methods for homogeneous ADCs.

## Methods

### Selection of sites to incorporate nnAA into trastuzumab

To select positions amenable to incorporation of the pAzF residue, we determined the surface accessibility of each residue of interest using the available crystal structures of the Fc^38^ fragment and Fab^39^ modeled on an intact IgG structure^40^ and 3D visualization within PyMol. 114 residues in HC that were surface-exposed were substituted *in silico* with phenylalanine to estimate the likely location of the azide functional group at the para position of the phenyl ring in the mutated protein.

### Preparation of DNA template

DNA constructs for the IgG variants were made incorporating a TAG amber codon at a defined position in the heavy or light chain of trastuzumab. Site directed mutagenesis was performed using a pYD plasmid containing the heavy chain coding region of trastuzumab with a C-terminal-(His)6 tag, or light chain coding region of trastuzumab only as DNA template and synthetic oligonucleotides (Eurofins MWS Operon; Huntsville, AL) containing mutations of interest in both sense and antisense directions.

### RF1 Strain Construction

The OMAR protocol was adapted from a previously reported protocol^21^. Briefly, SBJY001 containing the pKD46 plasmid^41^ were grown in 3 ml LB and 50 µg/mL ampicillin at 30 °C to OD_600_ 0.3. The cells were then induced with 1 mM L-arabinose at 37 °C for 45 min. The cell pellet was washed twice with cold 10% glycerol and resuspended in 30 µL cold 10% glycerol. 5 µM of each oligo was added to the resuspended cells. Synthetic oligos (Integrated DNA Technologies) were 90 base pairs long and designed to anneal to the lagging strand during DNA replication. The cells were electroporated at 1800 V for 5 ms in a 1 mm cuvette. They were then recovered in 3 mL LB and 50 µg/ml Amp. This process was repeated for 13 cycles. Cells were diluted and plated on LB agar plates and grown at 37 °C overnight. Colonies were screened using an adaptation of the Mismatch Amplification Mutation Assay (MAMA) PCR to identify positive hits^42^. Oligo sequences are listed in Table 2.

**Table 2:** Oligos for RF1 strain construction.

Oligo name	Oligo Sequence (5′ to 3′)
1opRF1 KR (OMAR)	GGGAAGTTGTAAGTACGGTTACGGTCGCTGCGATCcCCtgaaCCaAGacGcTTtCGACGGGTAGACGCTTCGGCCTGTTGGCGTTTTGCC
1opRF1 KRR (OMAR)	GGGAAGTTGTAAGTACGGTTACGGTCGCTGCGATCcCCtgaaCCacgacGcTTtCGACGGGTAGACGCTTCGGCCTGTTGGCGTTTTGCC
1opRF1 KRK (OMAR)	GGGAAGTTGTAAGTACGGTTACGGTCGCTGCGATCcCCtgaaCCcttacGcTTtCGACGGGTAGACGCTTCGGCCTGTTGGCGTTTTGCC
3KR op-PCR (MAMA)	GCG ATC CCC TGA ACC AAG ACG C
3 KRR op-PCR (MAMA)	CGATCcCCtgaaCCacgacGc
3KRK op-PCR (MAMA)	TGCGATCcCCtgaaCCcttacGc
5 RF1 op-PCR (MAMA)	CGTGACGGGGATAACGAACGCC

### Extract Preparation and Western Blot

Strains SBHS015, SBHS016 and SBHS017 were grown up in 500 mL TB at 37 °C shaking overnight in Tunair shake flasks. The cells were pelleted at 6000 × g for 15 minutes. The cell pellet was washed twice with 6 mL S30 Buffer (10 mM Tris, 14 mM Magnesium Acetate and 60 mM Potassium Acetate). The cells were then resuspended in 2 mL S30 Buffer for each 1 g cell pellet. The resuspended cells were lysed using a homogenizer. The extract was then clarified twice at 15,000 × g for 30 minutes. The extract was activated for 1, 2 or 3 hrs in a 30 °C water bath. An anti-RF1 antibody was generated by immunization of rabbits with purified recombinant *E. coli* RF1 protein followed by purification using an affinity matrix (YenZym Antibodies LLC). The specificity of the antibody was confirmed using ELISAs and Western Blots of the recombinant protein. The cell pellet, lysate and extract samples were run on a SDS-PAGE gel and transferred to a PVDF membrane using the iBlot system (Invitrogen). The primary anti-RF1 antibody was used followed by a secondary anti-rabbit alkaline-phosphatase conjugated antibody (Invitrogen). The bands were visualized using an alkaline-phosphatase chromogenic substrate solution containing 5-bromo-4-chloro-3-indolyl-1-phosphate and nitroblue tetrazolium (Invitrogen).

### Cell-free expression

The trastuzumab variant antibodies (trastuzumab^CF^) were expressed in a Xpress CF™ reaction as described previously^4^ with the modifications described below. The cell-free extracts used for this work were prepared from a mixture of two extracts derived from two engineered strains: 1) an OmpT sensitive RF1 attenuated *E. coli* strain engineered to overexpress *E. coli* DsbC^43^, and 2) a similar RF1 attenuated *E. coli* strain engineered to produce an orthogonal CUA-encoding tRNA non-natural amino acid insertion at an Amber Stop Codon. This cell-free extract blend (85%:15%) was treated with 50 µM iodoacetamide for 30 min at RT (20 °C) and added to a premix containing all other components, except for IgG heavy and light chain DNA. The final concentration in the protein synthesis reaction was 30% (v/v) cell extract, 2 mM para-azidophenylalanine (pAMF) (RSP Amino Acids), 5 uM engineered pAMF-specific amino-acyl tRNA synthetase (FRS variant), 2 mM GSSG, 8 mM magnesium glutamate, 10 mM ammonium glutamate, 130 mM potassium glutamate, 35 mM sodium pyruvate, 1.2 mM AMP, 0.86 mM each of GMP, UMP, and CMP, 2 mM amino acids (except 0.5 mM for Tyrosine and Phenylalanine), 4 mM sodium oxalate, 1 mM putrescine, 1.5 mM spermidine, 15 mM potassium phosphate, 100 nM T7 RNAP, 2 µg/mL trastuzumab light chain DNA, and 8 µg/mL trastuzumab-(His)6 heavy chain DNA. Cell-free reactions were initiated by addition of plasmid DNA of heavy and light chain and incubated at 30 °C for 12 h on a shaker at 650 rpm in 96-well plates at 100 uL scale or in 100 × 10 mm petri dish at 10 mL scale.

### ^14^C Autoradiogram and Suppression Determination

To quantify the suppression of amber codon, the reaction mixture was supplemented with a small amount of ^14 ^C labeled leucine (3 uL per 100 uL reaction, PerkinElmer: NEC279E001MC, 0.1 mCi/mL)^44^. The suppression of amber codon at different sites of the heavy or light chain was determined by [^14^C]-autoradiograhy of reducing SDS-PAGE gels. Full length trastuzumab heavy chain and suppressed trastuzumab heavy chain variants run at 50 kD on SDS-PAGE; whereas full length light chain and suppressed trastuzumab light chain runs at 25 kD. Non suppressed (truncated) trastuzumab variants run at a lower molecular weight. Amber suppression in the heavy is determined by:

1 $$suppression=\frac{band\,intensity\,of\,suppressed\,heavy\,or\,light\,chain\,TAG\,variant}{band\,intensity\,of\,wild\,type\,heavy\,or\,light\,chain}$$

Band intensity was determined by ImageQuant (Amersham Biosciences Corp.; Piscataway, NJ).

### Protein Purification

100 mL of crude cell- free material was clarified using centrifugation after 2:1 dilution with 100 mM sodium phosphate, 150 mM sodium chloride, pH 7.4. The resultant supernatant was then applied to a 1 mL HiTrap MabSelect SuRE (GE Healthcare Life Sciences; Piscataway, NJ) column for IgG capture according to the manufacturer’s recommendation. The IgG was eluted with 0.1 M citric acid, 300 mM L-arginine-HCl, pH 3.0, and the resulting pool adjusted to pH 4.6 with 1 M Tris, pH 9.0. Subsequently, this pool was processed on a 1 mL HiTrap Capto adhere (GE Healthcare Life Sciences; Piscataway, NJ) column that had previously been equilibrated with 0.1 M Tris-citrate, 300 mM L- arginine-HCl, pH 4.6. The IgG contained in the flow-through was collected and then buffer exchanged via dialysis into PBS.

### Drug Conjugation

Purified trastuzumab variants containing pAMF were conjugated to a cytotoxic agent, MMAF, using a strained cyclooctyne reagent. In brief, DBCO-MMAF (ACME Bioscience; Palo Alto, CA) was dissolved in DMSO to a final concentration of 5 mM. The compound was added to the purified protein sample in PBS buffer at the drug to antibody molar ratio of 5 to 1. Mixture was incubated at RT (20 °C) for 17 hours. Excess free drug was removed by Zeba plates (Thermo Scientific) equilibrated in PBS.

### Determination of Drug to Antibody Ratio by LC-MS

Spectra were acquired on an Agilent Technologies 6520 Accurate-Mass Q- TOF LC/MS system equipped with a 1200 series Binary SL pump and dual spray source. Water with 0.1% formic acid and a 20:80 isopropanol:acetonitrile solution with 0.1% formic acid were used as mobile phase A and B, respectively. Samples were separated over an PLRP-S polymeric reversed-phase column (50 × 2.1 mm, 5 μm, 4000 Å from Aglient Technologies) with loading at 0.2 mL/min in 10% mobile phase B followed by a 5 min on-column wash with the flow diverted to waste. Afterward at jump to 30%B over 1 min a 2.5%B/min gradient was run to 45%B, followed by a 5%B/min gradient to 60%B. The column was then cleaned at 95%B followed by three, 30 s sawtooth gradients between 95% and 10%B. Spectra were acquired at the slowest flow rate in high resolution mode with the Fragmentor set to 370 V.

Data were analyzed using MassHunter Qualitative. Mass spectra were combined over the entire elution range of all conjugates after determination of each conjugate’s elution profile as determined by an extracted ion chromatogram of its most abundant ions. Mass spectra were deconvoluted using the Maximum Entropy deconvolution algorithm. The DAR for all samples was determined as a weighted average of the decovoluted mass spectrum peak intensities for each conjugate.

### Thermofluor Assay

The Thermofluor assay (thermal shift assay) was carried out by mixing the protein to be assayed (trastuzumab^CF^ and variants thereof) with an environmentally sensitive dye (SYPRO Orange, Life Technologies Cat #S-6650) in a buffered solution (PBS), and monitoring the fluorescence of the mixture in real time as it undergoes controlled thermal denaturation. The final concentration of the protein in the assay mixture was between 100–250 µg/mL, and the dye was 1:1000 diluted from the original stock (Stock dye is 5000x in DMSO). After dispensing 5 µL aliquots of the protein-dye mixture in a 384-well microplate (Bio-Rad Cat #MSP-3852), the plate was sealed with an optically clear sealing film (Bio-Rad Cat #MSB-1001), and placed in a 384-well plate real-time thermocycler (Bio-Rad CFX384 Real Time System). The protein-dye mixture was heated from 25 °C to 95 °C, at increments of 0.1 °C per cycle (~1.5 °C per minute), allowing 3 seconds of equilibration at each temperature before taking a fluorescence measurement. At the end of the experiment, the melting temperature (Tm) was determined using the Bio-Rad CFX manager software. For protein samples with complex thermal transition profiles, the melting temperature (Tm) is calculated from the negative first-order derivative plot of fluorescence intensity (Y-axis) against temperature (X-axis), or by fitting the data to the Boltzmann sigmoidal model. The difference in melting temperature of IgG variants compared to the wild-type protein is a measure of the thermal shift for the protein being assayed.

### Cell Binding

Tumor cell lines (breast carcinoma BT-474, SKBR-3, JIMT-1, MDA-MB-468, MDA-MB-361) were obtained from American Type Culture Collection. The breast tumor line, KPL-4, was obtained from Professor J. Kurebayashi from Kawasaki Medical School, Kurashiki, Japan. Cells were maintained in Ham’s F-12: high glucose DMEM (50:50) (Cellgro-Mediatech; Manassas, VA) supplemented with 10% heat-inactivated fetal bovine serum (Hyclone; Thermo Scientific; Waltham, MA), 1% Penicillin/Streptomcin (Cellgro-Mediatech; Manassas, VA) and 2 mmol/L-glutamax (Life Technologies; Carlsbad, CA). Adherent cells were washed twice with calcium and magnesium-free Hanks Balanced Salt Solution (HBSS), harvested with HYQ®TASE™ (Hyclone; Thermo Scientific; Waltham, MA) and suspended in FACS buffer (DPBS buffer supplemented with 1% bovine serum albumin). A total of 200,000 cells per sample were incubated on ice for 60 mins with serial dilutions of trastuzumab^CF^ variants with or without conjugation, clinical grade Herceptin^®^ was used as a control. Cells were washed twice with ice-cold FACS buffer and incubated with 5 µg/ml Alexa 647 labeled goat anti-human IgG antibody (Life Technologies; Carlsbad, CA) on ice for 60 mins. Unstained cells, and cells stained with human IgG1 (Isotype control) or secondary antibody alone were used as controls. Samples were washed twice using FACS buffer and analyzed using a BD FACS Calibur system (BD Biosciences; San Jose, CA). Mean fluorescence intensities were fitted using non-linear regression analysis with one-site specific binding equation on GraphPad Prism (GraphPad v 5.00, Software; San Diego, CA). Data was expressed as Relative MFI vs. concentration of conjugated or un-conjugated antibody variants in nM.

### Cell Cytotoxicity Assay

Cytotoxicity effects of the conjugated trastuzumab^CF^ variants on cells were measured with a cell proliferation assay. SKBR3 and MDA-MB-468 cells (A total of 10e3 cells per well) were seeded in a volume of 40 μl in a 96-well half area flat bottom white polystyrene plate. The cells were allowed to adhere overnight at 37 °C in a CO_2_ incubator. ADC variants were formulated at 2x concentration in DMEM/F12 medium and filtered through MultiScreen HTS 96-Well Filter Plates (Millipore; Billerica, MA). Filter sterilized conjugated or unconjugated trastuzumab^CF^ variants were added into treatment wells and plates were cultured at 37 °C in a CO_2_ incubator for 120 hrs. For cell viability measurement, 80 μl of Cell Titer-Glo® reagent (Promega Corp.; Madison, WI) was added into each well, and plates processed as per product instructions. Relative luminescence was measured on an ENVISION® plate reader (Perkin-Elmer; Waltham, MA). Relative luminescence readings were converted to % viability using untreated cells as controls. Data was fitted with non-linear regression analysis, using log(inhibitor) vs. response, variable slope, 4 parameter fit equation on GraphPad Prism (GraphPad v 5.00, Software; San Diego, CA). Data was expressed as % relative cell viability vs. dose of ADC in nM.

### Her2 ECD ELISA

To measure total IgG concentration in plasma samples, 96-well ELISA plates were coated with 0.5 µg/mL HER2 ECD in carbonate/bicarbonate buffer (pH 9.6) at 4 °C overnight. After removal of the coat solution, nonspecific binding sites were blocked with blocking solution [0.5% bovine serum albumin (BSA) in PBS] for 1 to 2 hrs at RT. The plates were then washed with washing buffer (0.05% Tween in PBS), and standards or samples diluted in ELISA assay buffer [PBS containing 0.5% BSA, 0.05% Tween, 10 ppm proclin 300, 0.2% bovine g-globulin, 0.25% CHAPS, 0.35 mol/L NaCl, 5 mmol/L EDTA (pH 7.4)] were added. After a 2-h incubation, plates were washed and HRP conjugated goat anti-human Fc (Jackson ImmunoResearch Laboratories, West Grove, PA) was added and incubated at RT for an additional 2 h. Plates were then washed again, followed by the addition of tetramethyl benzidine substrate (KPL, Gaithersburg, ML) for color development. The reaction was stopped after 10 to 15 min by the addition of 1 mol/L phosphoric acid. Plates were read on a Molecular Devices microplate reader at a wavelength of 450 nm. The concentration of IgG in the samples was extrapolated from a four-variable fit of the standard curve.

### *In Vivo* Stability Study of ADC

The ADC drug-linker *in vivo* stability studies were run in Beige nude XID or Balb/c mice. Pharmacokinetic properties of antibodies observed in immune deficient background is similar to that observed for chimeric antibodies in immune competent and Balb/c mice^45^. All animal studies were performed within the guidelines of Institutional Animal Care and Use Committee Guidebook (2^nd^ Edition 2002, Reprinted 2008). The study protocols/procedures for these animal studies were approved by IACUC at Bayside Biosciences Inc. Animals were administered a single i.v. bolus dose of 2 or 5 mg/kg. Blood samples were collected as terminal bleeding at selected time points for up to 21 days. Blood samples were processed to obtain plasma, and stored at −80 °C until analysis. The total antibody concentrations in the plasma were quantified by Her2 ECD ELISA as described above and the DAR (drug antibody ratio) of the ADC were measured by a method using affinity capture followed by LCMS analysis. The method was as described^1^ with following key modifications: Streptavidin Mag Sepharose Beads (GE Healthcare, Cat#28-9857-99) were used to capture biotinylated (Fab)2 Goat Anti-Human IgG Fcγ fragment specific antibody (Jackson ImmunoResearch, West Grove, PA) or biotinylated Her2 ECD. Comparable results were observed with either biotinylated proteins. No detergent was used in the wash steps. No deglycosylation step was necessary, since the produced antibodies were aglycosylated. Eluted antibodies were neutralized immediately with 1 M Tris-HCl pH 9.0 buffer prior to LCMS analysis. The method established by Xu and colleagues^46^ was modified to improve sensitivity and simplified for our aglycosylated ADCs.

### *In Vivo* Efficacy Studies

KPL-4 cells were harvested with HYQ®TASE™ (Hyclone; Thermo Scientific; Waltham, MA), suspended in culture medium mixed 1:1 with phenol red–free Matrigel (Becton Dickinson Bioscience, San Jose, CA). 3 million of the mixed KPL4 cells were inoculated in the mammary fat pad of naive female Beige nude XID mice. All animals were randomly assigned into treatment groups, such that the mean tumor volume for each group was roughly 300 mm^3^. Trastuzumab^CF^ or trastuzumab^CF^ drug conjugates were given by a single i.v. injection at treatment day 0. Vehicle control was either PBS or free drug dosed at equal drug exposure calculated based on ADC dose. All treatment groups consisted of 10 animals per group, and tumor size was monitored twice a week using caliper measurement. Mice were housed in standard rodent micro-isolator cages. Environmental controls for the animal rooms were set to maintain a temperature of ~70 °F, a relative humidity of 40% to 60%, and an approximate 14-h light/10-h dark cycle. Tumor volume (mm^3^) and body weights (g) were measured every 2 days. Animals studies were performed within the guidelines of Institutional Animal Care and Use Committee Guidebook (2^nd^ Edition 2002, Reprinted 2008).

## Electronic supplementary material

Supplementary info
