# Supplementary material for: RF1 attenuation enables efficient non-natural amino acid incorporation for production of homogeneous antibody drug conjugates
Source: Sci Rep. 2017 Jun 8;7:3026. doi: 10.1038/s41598-017-03192-z (PMC5465077; doi:10.1038/s41598-017-03192-z)
Supplement: Supplementary file 1 — Supplementary info [file 41598_2017_3192_MOESM1_ESM.doc]

**RF1 attenuation enables efficient non-natural amino acid incorporation for production of homogeneous antibody drug conjugates**

Gang Yin*, Heather Stephenson*, Junhao Yang, Xiaofan Li, Stephanie Armstrong, Tyler H. Heibeck, Cuong Tran, Mary Rose Masikat, Sihong Zhou, Ryan L. Stafford, Alice Y. Yam, John Lee, Alexander R. Steiner, Avinash Gill, Kalyani Penta, Sonia Pollitt, Ramesh Baliga, Christopher J. Murray, Christopher D. Thanos, Leslie M. McEvoy, Aaron K. Sato and Trevor J. Hallam

Sutro Biopharma Inc, South San Francisco, CA 94080 USA

*These authors contributed equally to this work.

Correspondence should be addressed to G.Y. (gyin@sutrobio.com) and T.J.H. (thallam@sutrobio.com)

Keywords: antibody-drug conjugate, cell-free protein synthesis, RF1

Table S1a Suppression of TAG sites in HC

| position | suppression | position | suppression | position | suppression |
| --- | --- | --- | --- | --- | --- |
| V005 | 75% | V264 | 19% | I332 | 24% |
| A022 | 32% | D265 | 21% | E333 | 24% |
| G042 | 48% | S267 | 4% | K334 | 55% |
| G065 | 26% | H268 | 61% | T335 | 41% |
| S074 | 53% | E269 | 14% | S337 | 12% |
| A084 | 51% | D270 | 18% | A339 | 15% |
| A118 | 36% | P271 | 18% | K340 | 4% |
| S119 | 43% | E272 | 17% | G341 | 1% |
| S131 | 13% | K274 | 9% | Q342 | 1% |
| S132 | 57% | F275 | 30% | P343 | 1% |
| S134 | 34% | Y278 | 42% | R344 | 5% |
| T135 | 38% | D280 | 11% | R355 | 9% |
| S136 | 38% | G281 | 20% | E356 | 7% |
| G137 | 42% | V282 | 27% | M358 | 4% |
| G138 | 42% | E283 | 9% | T359 | 14% |
| T139 | 34% | H285 | 16% | K360 | 9% |
| T155 | 48% | N286 | 14% | N361 | 2% |
| S160 | 45% | T289 | 46% | Q362 | 3% |
| G161 | 31% | R292 | 28% | K370 | 9% |
| A162 | 50% | E293 | 19% | Y373 | 1% |
| T164 | 17% | E294 | 4% | S375 | 13% |
| S165 | 64% | Q295 | 4% | W381 | 4% |
| A172 | 57% | Y296 | 17% | S383 | 9% |
| L174 | 18% | N297 | 1% | N384 | 34% |
| S176 | 57% | S298 | 6% | Q386 | 4% |
| S177 | 22% | T299 | 1% | N389 | 10% |
| S191 | 42% | Y300 | 4% | Y391 | 10% |
| G194 | 43% | R301 | 4% | K392 | 2% |
| T195 | 9% | V303 | 14% | L398 | 5% |
| T197 | 13% | V305 | 4% | S400 | 6% |
| S219 | 11% | K317 | 4% | F404 | 1% |
| P238 | 22% | K320 | 1% | F405 | 2% |
| S239 | 56% | K322 | 17% | S415 | 1% |
| F241 | 30% | S324 | 24% | Q418 | 1% |
| F243 | 24% | K326 | 9% | G420 | 4% |
| K246 | 46% | A327 | 8% | N421 | 4% |
| T260 | 5% | P329 | 13% | V422 | 0% |
| V262 | 20% | A330 | 18% | S424 | 11% |

Table S1b Suppression of TAG sites in LC

| position | suppression | position | suppression | position | suppression |
| --- | --- | --- | --- | --- | --- |
| LC--1 | 59% | LC-R66 | 10% | LC-K145 | 26% |
| LC-D1 | 29% | LC-T69 | 32% | LC-Q147 | 6% |
| LC-Q3 | 35% | LC-D70 | 31% | LC-K149 | 31% |
| LC-T5 | 20% | LC-T72 | 34% | LC-V150 | 12% |
| LC-S7 | 11% | LC-T74 | 38% | LC-D151 | 18% |
| LC-P8 | 10% | LC-S76 | 26% | LC-N152 | 36% |
| LC-S9 | 20% | LC-S77 | 12% | LC-L154 | 6% |
| LC-S10 | 29% | LC-Q79 | 17% | LC-Q155 | 28% |
| LC-L11 | 33% | LC-P80 | 72% | LC-N158 | 35% |
| LC-S12 | 31% | LC-E81 | 84% | LC-S159 | 8% |
| LC-S14 | 16% | LC-T85 | 17% | LC-E161 | 10% |
| LC-G16 | 8% | LC-T97 | 77% | LC-V163 | 18% |
| LC-D17 | 2% | LC-Q100 | 10% | LC-E165 | 26% |
| LC-R18 | 33% | LC-K103 | 10% | LC-D167 | 14% |
| LC-V19 | 7% | LC-E105 | 40% | LC-K169 | 38% |
| LC-T20 | 10% | LC-I106 | 44% | LC-D170 | 28% |
| LC-T22 | 2% | LC-K107 | 4% | LC-S171 | 42% |
| LC-R24 | 4% | LC-R108 | 10% | LC-T172 | 5% |
| LC-A25 | 38% | LC-A111 | 52% | LC-Y173 | 75% |
| LC-S26 | 30% | LC-F116 | 24% | LC-L175 | 24% |
| LC-Q27 | 63% | LC-S121 | 40% | LC-S177 | 23% |
| LC-Q37 | 5% | LC-D122 | 13% | LC-T180 | 24% |
| LC-K39 | 3% | LC-E123 | 6% | LC-L181 | 23% |
| LC-P40 | 3% | LC-L125 | 2% | LC-S182 | 21% |
| LC-K42 | 15% | LC-K126 | 14% | LC-K183 | 71% |
| LC-K45 | 27% | LC-G128 | 5% | LC-D185 | 19% |
| LC-S52 | 4% | LC-T129 | 48% | LC-Y186 | 42% |
| LC-Y55 | 4% | LC-N137 | 48% | LC-E187 | 32% |
| LC-V58 | 3% | LC-N138 | 14% | LC-K188 | 36% |
| LC-P59 | 57% | LC-Y140 | 15% | LC-H189 | 13% |
| LC-R61 | 58% | LC-P141 | 3% | LC-K190 | 67% |
| LC-S63 | 16% | LC-R142 | 21% | LC-A193 | 26% |
| LC-S65 | 6% | LC-E143 | 20% | LC-E195 | 27% |

**Table S2a Suppression of TAG sites in HC in RF-1 attenuated extract**

| position | suppression | position | suppression | position | suppression |
| --- | --- | --- | --- | --- | --- |
| HC--1 | 67% | HC-D72 | 32% | HC-G166 | 61% |
| HC-E1 | 68% | HC-T73 | 40% | HC-V167 | 57% |
| HC-Q3 | 73% | HC-K75 | 72% | HC-T169 | 62% |
| HC-S7 | 50% | HC-T77 | 69% | HC-G178 | 31% |
| HC-G8 | 43% | HC-Y79 | 51% | HC-L179 | 59% |
| HC-G9 | 42% | HC-Q81 | 54% | HC-Y180 | 44% |
| HC-G10 | 38% | HC-N82A | 48% | HC-S184 | 56% |
| HC-Q13 | 56% | HC-S82B | 73% | HC-V186 | 77% |
| HC-P14 | 63% | HC-R83 | 100% | HC-T187 | 74% |
| HC-G15 | 55% | HC-E85 | 53% | HC-P189 | 56% |
| HC-G16 | 50% | HC-T87 | 62% | HC-S190 | 63% |
| HC-S17 | 100% | HC-V89 | 70% | HC-S192 | 66% |
| HC-L18 | 53% | HC-Y91 | 58% | HC-L193 | 57% |
| HC-R19 | 100% | HC-R94 | 71% | HC-Q196 | 62% |
| HC-S21 | 100% | HC-G97 | 83% | HC-N203 | 64% |
| HC-S25 | 100% | HC-D98 | 48% | HC-K205 | 75% |
| HC-G26 | 100% | HC-F100 | 33% | HC-S207 | 92% |
| HC-N28 | 100% | HC-Y102 | 67% | HC-N208 | 67% |
| HC-K30 | 100% | HC-Q105 | 54% | HC-T209 | 73% |
| HC-T32 | 57% | HC-L108 | 83% | HC-K210 | 68% |
| HC-A40 | 72% | HC-T110 | 89% | HC-V211 | 77% |
| HC-G42 | 51% | HC-S112 | 90% | HC-D212 | 34% |
| HC-K43 | 68% | HC-S113 | 48% | HC-K213 | 63% |
| HC-Y52 | 74% | HC-T120 | 58% | HC-K214 | 41% |
| HC-P52A | 100% | HC-K121 | 57% | HC-E216 | 72% |
| HC-T53 | 100% | HC-G122 | 24% | HC-P217 | 84% |
| HC-N54 | 100% | HC-F126 | 100% | HC-K218 | 100% |
| HC-G55 | 46% | HC-P127 | 87% | HC-D221 | 75% |
| HC-Y56 | 95% | HC-K133 | 70% | HC-K222 | 87% |
| HC-T57 | 100% | HC-A140 | 97% | HC-T223 | 82% |
| HC-Y59 | 100% | HC-K147 | 79% | HC-T225 | 81% |
| HC-D61 | 100% | HC-D148 | 34% | HC-P227 | 68% |
| HC-S62 | 100% | HC-F150 | 75% | HC-P228 | 85% |
| HC-K64 | 100% | HC-E152 | 65% | HC-P230 | 100% |
| HC-R66 | 23% | HC-S157 | 74% | HC-A231 | 97% |
| HC-T68 | 80% | HC-N159 | 72% | HC-G236 | 70% |
| HC-S70 | 38% | HC-L163 | 61% | HC-G237 | 100% |

**Table S2b Suppression of TAG sites in L**C in RF-1 attenuated extract

| position | suppression | position | suppression | position | suppression |
| --- | --- | --- | --- | --- | --- |
| LC--1 | 66% | LC-S76 | 70% | LC-S159 | 86% |
| LC-D1 | 72% | LC-S77 | 67% | LC-V163 | 83% |
| LC-Q3 | 66% | LC-Q79 | 60% | LC-E165 | 75% |
| LC-T5 | 72% | LC-P80 | 64% | LC-D167 | 86% |
| LC-S7 | 77% | LC-E81 | 56% | LC-D170 | 100% |
| LC-P8 | 71% | LC-T85 | 64% | LC-S171 | 100% |
| LC-S10 | 76% | LC-T97 | 67% | LC-T172 | 82% |
| LC-L11 | 71% | LC-Q100 | 75% | LC-Y173 | 100% |
| LC-S12 | 71% | LC-E105 | 100% | LC-L175 | 94% |
| LC-S14 | 72% | LC-I106 | 74% | LC-S177 | 85% |
| LC-G16 | 68% | LC-K107 | 78% | LC-T180 | 100% |
| LC-D17 | 59% | LC-R108 | 60% | LC-L181 | 85% |
| LC-R18 | 67% | LC-A111 | 72% | LC-S182 | 100% |
| LC-V19 | 63% | LC-F116 | 84% | LC-K183 | 82% |
| LC-T20 | 83% | LC-S121 | 68% | LC-D185 | 100% |
| LC-R24 | 66% | LC-D122 | 74% | LC-Y186 | 100% |
| LC-A25 | 62% | LC-E123 | 70% | LC-E187 | 100% |
| LC-S26 | 61% | LC-K126 | 83% | LC-K188 | 100% |
| LC-Q27 | 70% | LC-G128 | 34% | LC-H189 | 100% |
| LC-Q37 | 67% | LC-T129 | 79% | LC-A193 | 80% |
| LC-K39 | 62% | LC-N137 | 61% | LC-E195 | 100% |
| LC-P40 | 65% | LC-N138 | 57% | LC-T197 | 100% |
| LC-K42 | 62% | LC-Y140 | 78% | LC-Q199 | 71% |
| LC-K45 | 66% | LC-P141 | 27% | LC-G200 | 58% |
| LC-Y55 | 69% | LC-R142 | 51% | LC-L201 | 100% |
| LC-V58 | 71% | LC-E143 | 71% | LC-P204 | 100% |
| LC-P59 | 84% | LC-Q147 | 65% | LC-K207 | 100% |
| LC-R61 | 76% | LC-K149 | 72% | LC-S208 | 100% |
| LC-S63 | 85% | LC-V150 | 45% | LC-R211 | 100% |
| LC-S65 | 83% | LC-D151 | 24% | LC-G212 | 100% |
| LC-R66 | 93% | LC-N152 | 87% | LC-E213 | 100% |
| LC-T69 | 84% | LC-L154 | 69% | LC-C214 | 86% |
| LC-D70 | 73% | LC-Q155 | 64% |  |  |
| LC-T74 | 80% | LC-N158 | 84% |  |  |

**Table S3. Comparison of *in vivo*** efficacy differences at day 45

| Dunnett's multiple comparisons test | Significant? | Summary | Adjusted P Value |
| --- | --- | --- | --- |
| IgG HC-F404 AB4285, 15mg/kg vs. IgG HC-R355 AB4285, 15mg/kg | No | ns | 0.9271 |
| IgG HC-F404 AB4285, 15mg/kg vs. IgG HC-S136 AB4285, 15mg/kg | No | ns | > 0.9999 |
| IgG HC-F404 AB4285, 15mg/kg vs. IgG HC-S136, 15mg/kg | Yes | * | 0.0492 |
| IgG HC-F404 AB4285, 15mg/kg vs. HC N389 AB4285 15mg/kg | No | ns | 0.9365 |
| IgG HC-F404 AB4285, 15mg/kg vs. Vehicle | Yes | **** | < 0.0001 |
| IgG HC-F404 AB4285, 15mg/kg vs. Free Drug | Yes | *** | 0.0005 |
| IgG HC-R355 AB4285, 15mg/kg vs. IgG HC-F404 AB4285, 15mg/kg | No | ns | 0.9271 |
| IgG HC-R355 AB4285, 15mg/kg vs. IgG HC-S136 AB4285, 15mg/kg | No | ns | 0.943 |
| IgG HC-R355 AB4285, 15mg/kg vs. IgG HC-S136, 15mg/kg | Yes | ** | 0.0045 |
| IgG HC-R355 AB4285, 15mg/kg vs. HC N389 AB4285 15mg/kg | No | ns | 0.3924 |
| IgG HC-R355 AB4285, 15mg/kg vs. Vehicle | Yes | **** | < 0.0001 |
| IgG HC-R355 AB4285, 15mg/kg vs. Free Drug | Yes | **** | < 0.0001 |

**Table S4. Comparison of *in vivo*** efficacy differences at day 63

| Dunnett's multiple comparisons test | Significant? | Summary | Adjusted P Value |
| --- | --- | --- | --- |
| IgG HC-R355 AB4285, 15mg/kg vs. IgG HC-F404 AB4285, 15mg/kg | No | ns | 0.3676 |
| IgG HC-R355 AB4285, 15mg/kg vs. IgG HC-S136 AB4285, 15mg/kg | No | ns | 0.3926 |
| IgG HC-R355 AB4285, 15mg/kg vs. HC N389 AB4285 15mg/kg | Yes | * | 0.0346 |

**
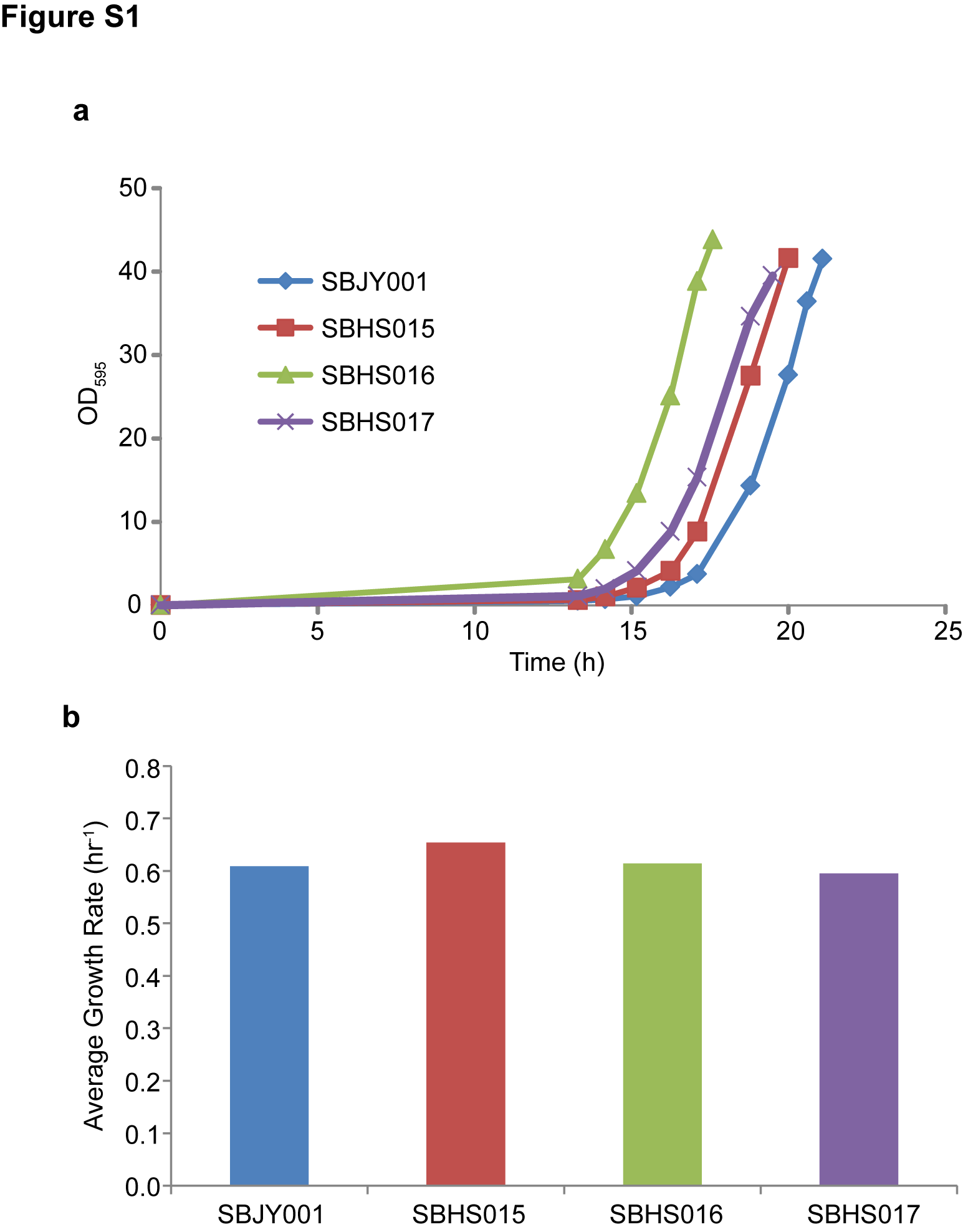
**

**Fig S1 RF1 mutant strains show equivalent growth to RF1 wt.** (a) Growth curves for RF1 mutant strains (SBHS015, SBHS016, and SBHS017) have similar log-phase growth rates to RF1 wild-type strain (SBJY001). (b) Average growth rates for RF1 mutant strains are equivalent to RF1 wild-type strain.

**
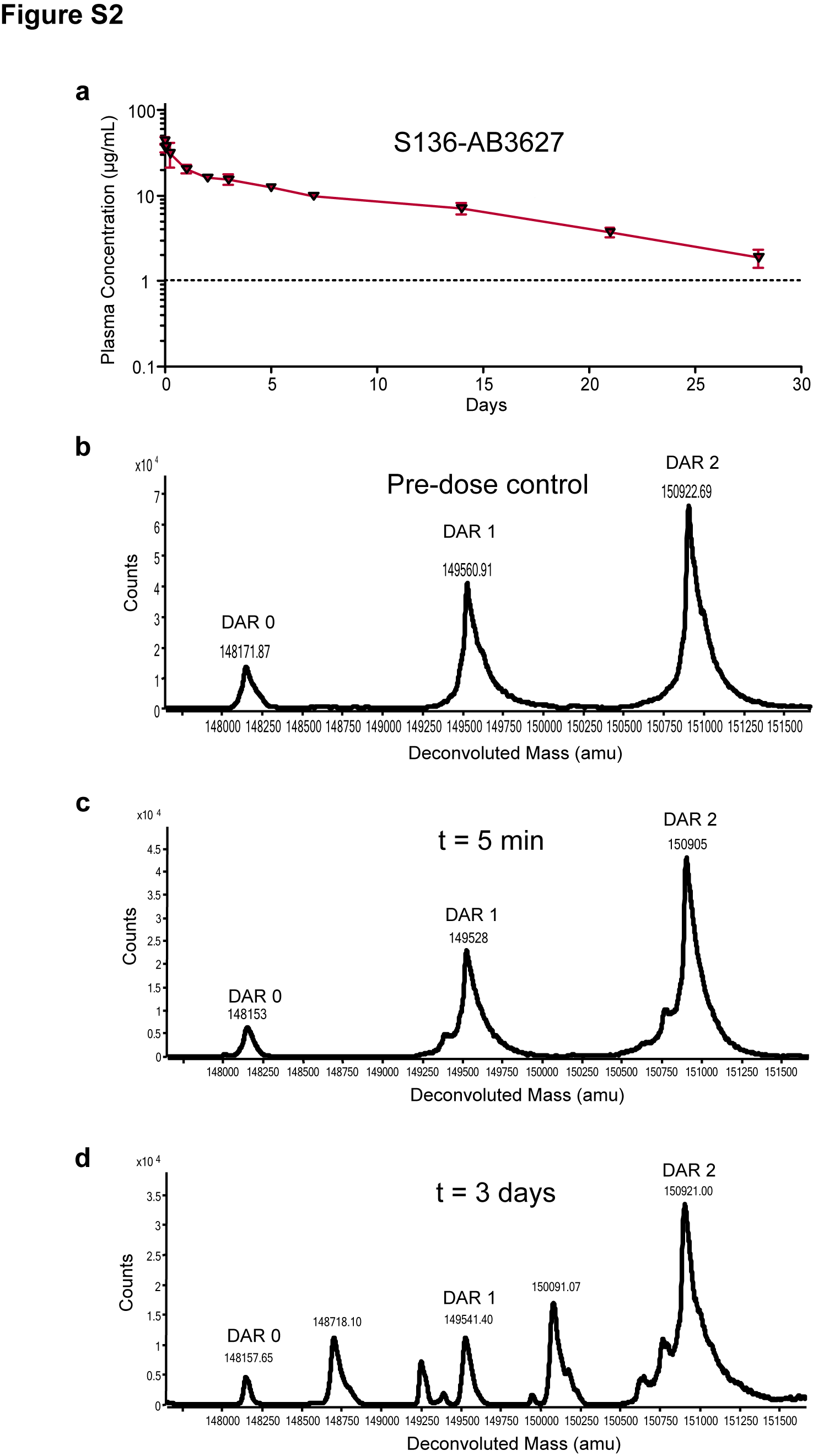
**

**Fig S2 Trastuzumab S136-AB3627 degrades in vivo.** (a) PK profile measuring antibody concentration in Balb/c after injection of 2 mg/kg ADC. Degraded linkers are not detectable by ELISA assay used to measure antibody concentration. (b) Pre-dose control LC/MS shows good resolution of DAR 0, 1, and 2 species in the ADC. (c) Immunoaffinity capture followed by LC/MS shows minor degradation of the ADC immediately post injection. (d) Immunoaffinity capture followed by LC/MS shows extensive degradation several days post injection consistent with drug-linker instability.

**Figures S3**


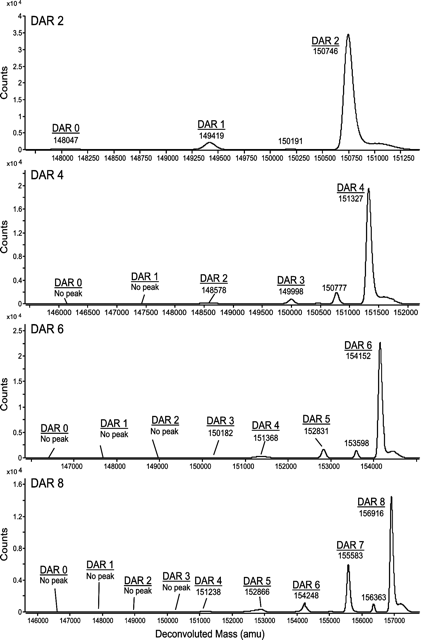


Figure S3: Intact deconvoluted spectra for ADCs containing 2, 4, 6, and 8 non-native amino acids labeled by their theoretical maximum DAR. Each spectrum is scaled to the minimum (no conjugated) and maximum (full conjugation) theoretical mass. “No peak” designates were signal within a reasonable mass difference of the theoretical mass was not observed.
